# A simple synthesis method for nanostructured Co-WC/carbon composites with enhanced oxygen reduction reaction activity

**DOI:** 10.1080/14686996.2016.1140305

**Published:** 2016-03-09

**Authors:** Jun Kang, Hye-min Kim, Nagahiro Saito, Myeong-Hoon Lee

**Affiliations:** ^a^ Division of Marine Engineering, Korea Maritime and Ocean University, Busan606-791, Republic of Korea; ^b^ Graduate School of Engineering, Nagoya University, Nagoya464-8603, Japan; ^c^ Department of Material Science and Engineering, Nagoya University, Furo-cho, Chikusa-ku, Nagoya464 8603, Japan

**Keywords:** Solution plasma processing, nanoparticle-carbon composite, oxygen reduction reaction, non-precious metal electrocatalysts

## Abstract

Co nanoparticles (Co NPs) and nanoscale tungsten carbide (WC) are successfully synthesized simultaneously with mesoporous structured carbon black (C) using an innovative simple method, which is known as solution plasma processing (SPP), and NPs are also loaded onto carbon black at the same time by SPP. The introduction of Co NPs led to not only superior oxygen reduction reaction (ORR) activity in terms of onset potential and peak potential, but also to a more efficient electron transfer process compared to that of pure WC. Co-WC/C also showed durability for long-term operation better than that of commercial Pt/C. These results clearly demonstrate that the presence of Co NPs significantly enhanced the ORR and charge transfer number of neighboring WC NPs in ORR activities. In addition, it was proved that SPP is a simple method (from synthesis of NPs and carbon black to loading on carbon black) for the large-scale synthesis of NP-carbon composite. Therefore, SPP holds great potential as a candidate for next-generation synthetic methods for the production of NP-carbon composites.

## Graphical abstract

**Figure:**
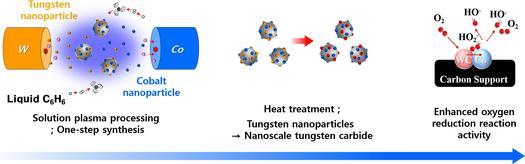

## 1. Introduction

The oxygen reduction reaction (ORR) is an important electrochemical reaction in many fields, including energy conversion (fuel cells, metal–air batteries), corrosion, biosensing and the production of hydrogen peroxide [1−4]. In particular, for fuel cell systems, the cathodic oxygen reduction is a major factor limiting performance because it is always a slow reaction in both acidic and basic solutions [5]. Currently the best and most frequently used catalyst for ORR is platinum because of its excellent ORR activity (i.e. low overpotential and large current density) and selectivity toward a direct four-electron pathway in both acidic and alkaline solutions. Aside from its activity, however, the Pt catalyst is disadvantageous in terms of its prohibitively high cost and limited supply, and its susceptibility to contamination by carbon monoxide (CO) [6]. As a result, the discovery of less expensive and more CO-tolerant alternatives to Pt electrocatalysts would greatly facilitate the commercialization of fuel cell systems. One of the most attractive ideas is the development of efficient non-precious metal electrocatalysts. Tungsten carbide (WC) is the most attractive material due to its high electrical conductivity, CO resistance, and corrosion resistance; it also has a platinum-like behavior for the chemisorption of hydrogen and oxygen [7−12]. However, the intrinsic catalysis of WC itself is very much lower than that of platinum, and thus its large over-potential for ORR must be reduced in order to obtain high energy efficiency. In addition, the methods that can be used to obtain nanoscale WC are complicated and generally require multiple step processes, high temperature, long processing time, and a chemical agent. Therefore, it is necessary to find a simple method to synthesize WC with good dispersion.

To the best of our knowledge, there have been few attempts to enhance the catalytic activity of WC based materials for ORR with non-noble metals. Herein, accordingly, for improved catalytic potential and durability of a cathode, a combination of nanoscale WC with cobalt (Co) nanoparticles is considered as a potential alternative electrode material for fuel cell application. Recently Co based catalysts have been studied as non-noble metals to enhance ORR activity of carbonaceous materials, for economic reasons, and showed significantly improved electro-catalytic activity of ORR [13−15].

In addition, in this work, a simple and effective method to synthesize highly dispersed nanoscale Co-WC on carbon materials (Co-WC/C) in the form of nanocomposites using a one-step process (from synthesis of nanoparticles and carbon black to loading on carbon) by solution plasma processing (SPP) was reported. SPP has been applied for synthesis of carbon black directly from hydrocarbon solution and metal nanoparticles by electrode sputtering process of the working electrode within short processing time at atmospheric pressure. [16−20]. Moreover, synthesized metal nanoparticles can be uniformly dispersed on carbon black because the synthesis and loading processes can occur at the same time.

In this study the physical and chemical characteristics of the obtained Co-WC/C are investigated. Then, the enhancement of catalytic activity due to the presence of Co within the WC/carbon matrix is discussed in detail. The durability of Co-WC/C is compared to that of commercial Pt/C.

## 2. Experimental procedures

### 2.1. Synthesis of Co-WC/C

**Figure 1.**
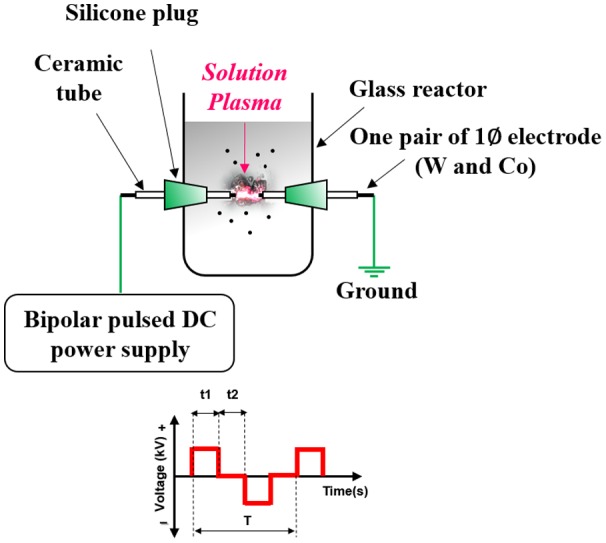
Schematic of the solution plasma process (SPP).

The experimental setup of SPP used for the production of Co-WC/C is schematically shown in Figure 1. Metal wires (W, Co) with diameter of 1.0 mm (W, Co, 99.95% Nilaco, Tokyo, Japan) and benzene (C_6_H_6_, 99.5% Kanto Chemical, Tokyo, Japan) were applied, respectively, as the precursors of Co, WC and carbon nanoparticles. To concentrate energy, each electrode was insulated by ceramic tubes; protruding length was 1.5 mm from the ceramic tube tips and tubes were inserted into a silicone plug. One pairs of metal electrodes (W, Co) was placed in a glass vessel (100 ml beaker, with a diameter of 5 cm and height of 7 cm); the distance between the tips of the electrodes was set at 0.5 mm. The electrodes were discharged in benzene using a bipolar–DC pulse power supply (Kurita, Kurita, Japan); the voltage, pulse frequency, and pulse width were controlled at 1.6 kV, 25 kHz, and 0.5 μs, respectively. Heat treatment was required to increase the conductivity of the carbon matrix and to facilitate the conversion from meta-stable carbide (cubic WC_1−X_) to carbon rich carbide (hexagonal WC); this process was performed in a tube furnace with a 30 min dwell time, under a flowing Ar atmosphere, with a heat treatment temperature (HTT) of 1100°C (heating rate: 25°C min^−1^ and cooling rate: 7°C min^−1^).

### 2.2. Characterization

Transmission electron microscopy (TEM, JEOL, JEM2500SE at 200 kV, JEOL Ltd., Tokyo, Japan) observations were conducted to study the microstructure, shape and size of the synthesized Co-WC/C nanocomposite. X-ray diffraction patterns (XRD, Rigaku Smart Lab Rigaku Corporation, Tokyo, Japan) were obtained using Cu Kα (*λ* = 1.54 nm) as a target over a scan range of 15–90° with 0.02° step size and 2° min^−1^ scan speed. The specific surface area, total pore volume, and mean pore size of all of the samples were calculated by N_2_ adsorption–desorption isotherms using the Brunauer–Emmett–Teller (BET) method (Belsorp-mini II, Belsorp, USA). All samples were degassed at 200°C for 2 h prior to measurement of the BET.

### 2.3. Electrode preparation and electrochemical characterization

In order to evaluate the electrochemical properties of Co-WC/C, three electrode cells with a potentiostat (Hokuto Denko, Inc., HZ5000, Tokyo, Japan) were applied. The three-electrode cell consisted of a glassy carbon (GC) disk (3 mm in diameter) working electrode, Ag/AgCl (saturated KCl) reference electrode, and a platinum wire counter electrode. The samples were prepared by ultrasonicating a mixture of 5 mg of sample powder, 0.5 ml of ethanol, and 50 μl of Nafion solution (Sigma Aldrich, Germany 5 wt% Nafion) until a homogeneous suspension was formed. A droplet (10 μl) of the obtained suspension was placed on the glassy carbon and dried at room temperature prior to the measurement.

The cyclic voltammetry (CV) method was conducted in 0.1 M KOH electrolyte and O_2_ gas was purged in the solution at a 50 mV s^−1^ scan rate from 0.3 V to –1 V (V versus Ag/AgCl). Moreover, we investigated the electron transfer kinetics during the ORR, and the linear sweep voltammetry (LSV) method was performed in 0.1 M KOH electrolyte and O_2_ gas was purged in the solution with a 10 mV s^−1^ scan rate from 0.2 V to –1 V (V versus Ag/AgCl) at 900 to 3600 rpm rotation speed using a rotating disk electrode (RDE). A commercial 20 wt% Pt/C catalyst was selected as a performance benchmark. The electrochemical durability was measured in chronoamperometric responses in 0.1 M KOH with saturated oxygen concentration for 20,000 s at –0.4 V.

## 3. Results and discussion

**Figure 2.**
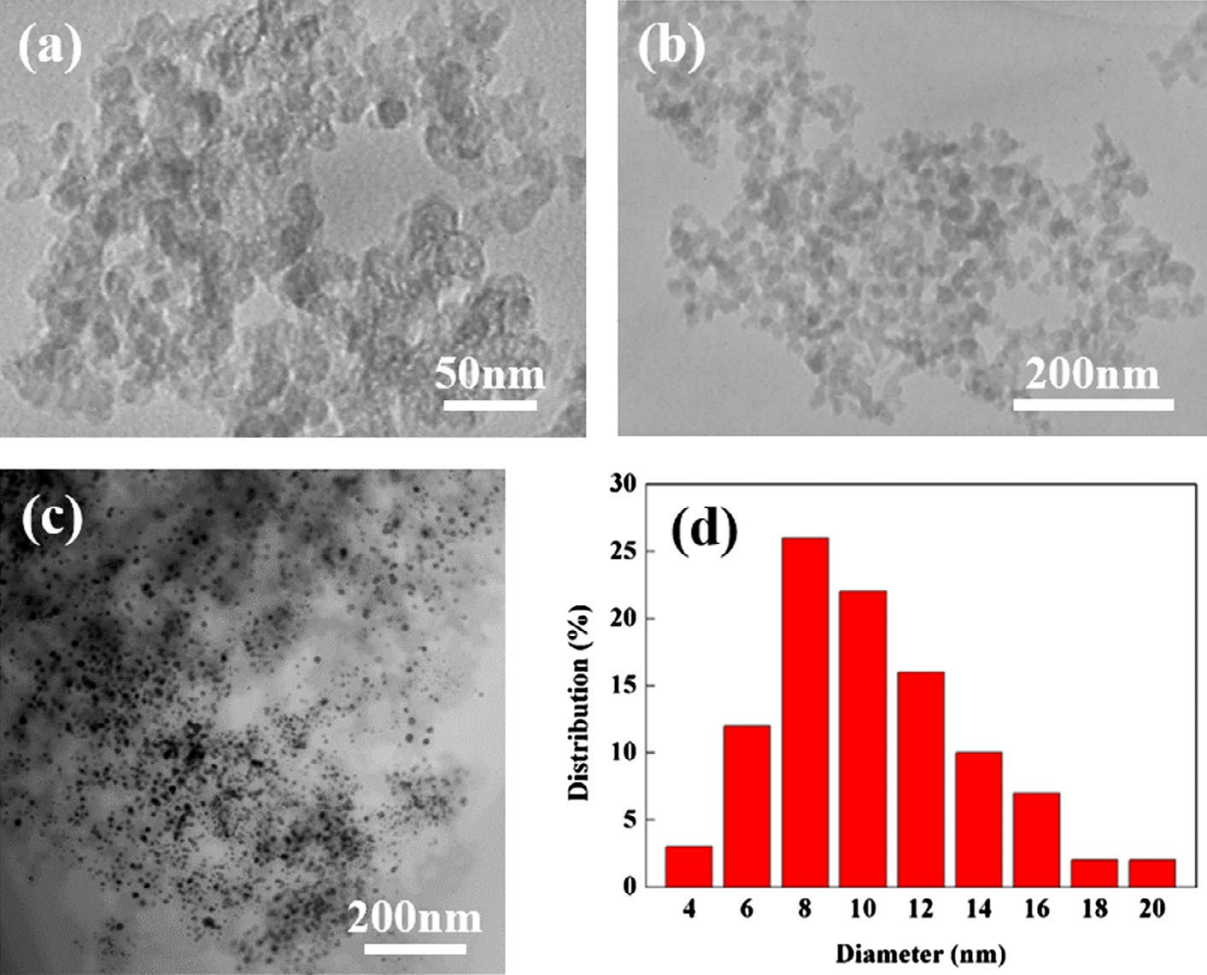
(a) High magnification TEM image of Co-WC/C; (b) low magnification TEM image Co-WC/C; (c) BF-STEM image of Co-WC/C; (d) size distribution of annealed Co-WC/C.

**Figure 3.**
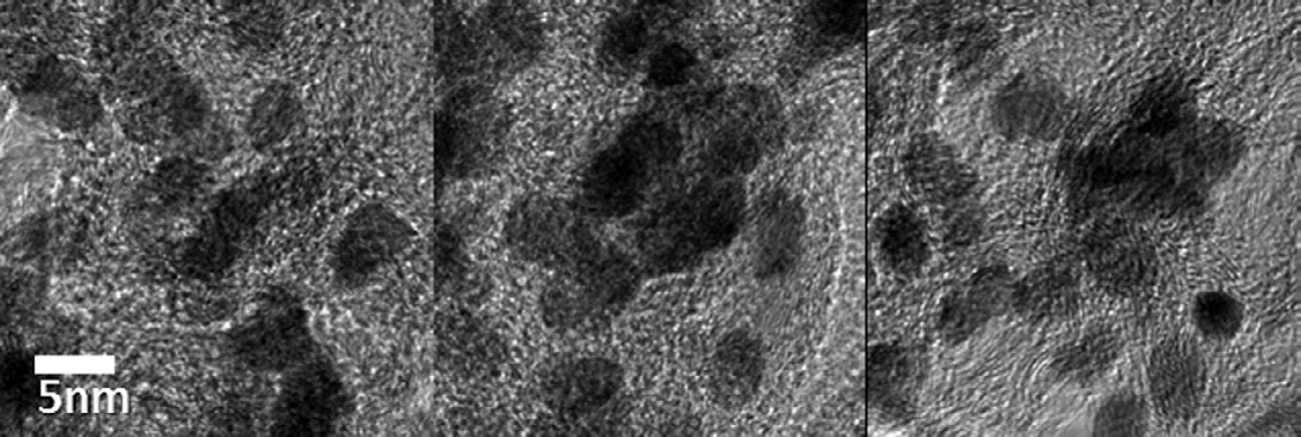
HR–TEM images of Co-WC/C.

**Figure 4.**
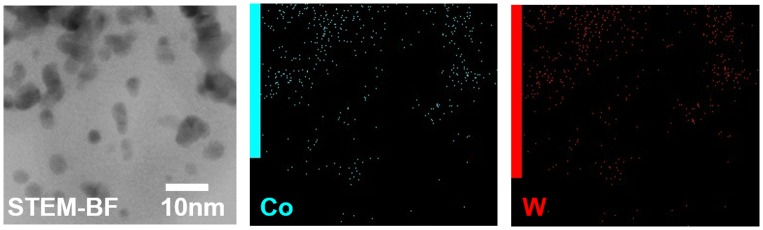
STEM–BF and STEM–EDX mapping images of Co-WC/C.

Approximately 1000 mg of Co-WC/C was generated from one pair of electrodes in 100 ml of benzene after 30 min of SPP. The microstructure of the Co-WC/C was observed using TEM, as shown in Figure 2(a) and (b). These images show that the carbons were spherical-like in shape, with a uniform size ranging from 20 to 30 nm; the carbons were found to exist in a chain-like morphology. The principle that explains how the carbons can agglomerate was discussed in previous studies [16–18]. The morphology of the Co-WC was determined using bright field scanning TEM (BF–STEM) images (Figure 2(c)) and high resolution TEM (HR–TEM) images (Figure 3). These images clearly demonstrate that the nearly spherical nanoparticles (NPs) exist stably without any aggregation. The images also clearly reveal that the NPs were remarkably uniform, and were well dispersed over the entire surface of the carbons. The images show that the NPs had a narrow size distribution, with an average size of 10 nm (Figure 2(d)). The STEM energy-dispersive X-ray spectroscopy (STEM–EDX) mapping images show that Co and WC NPs are uniformly distributed on carbon surface and both NPs adjoin each other (Figure 4).

Inductively coupled plasma-atomic emission spectrometry (ICPAES, Perkin Elmer, USA, Optima 3300DV) was applied for a qualitative and quantitative chemical analysis of the composites. The elemental composition of Co and W in the sample was calculated as 57.97 and 43.03%, respectively and the weight percentage of entire metal NPs in composite was 14.01%.

**Figure 5.**
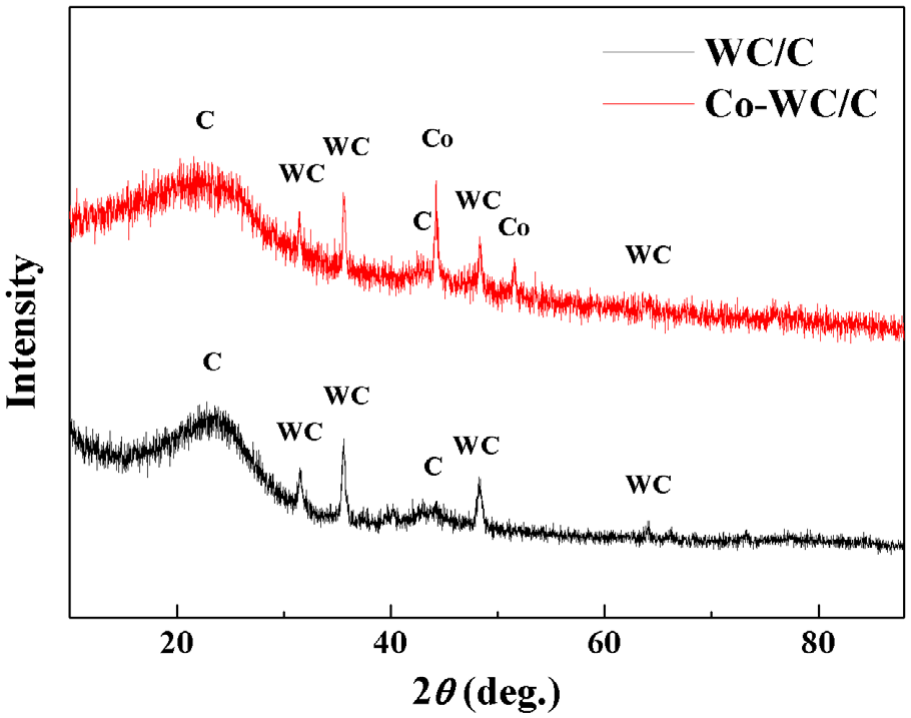
X-ray diffraction patterns for NP/C.

Figure 5 shows the XRD patterns of the nanocomposites synthesized from different metal electrodes. As can be seen in the spectra, five peaks were observed from all of samples, corresponding to the 111, 200, 220, 311, and 222 reflections of the planes of WC (JCPDS #25-1047); other small peaks were also detected that indicated the formation of Co NPs (JCPDS #15-0806) from the electrodes. Based on the peak areas from the XRD pattern, the average particle size was calculated using Scherrer’s formula *d* = 0.9*λ/β*cos*θ*, where 0.9 is the shape factor generally taken for a cubic system, *λ* is the X-ray source wavelength, which is typically 0.154 nm, *β* is the full width at half maximum intensity in radians, and *θ* is the Bragg angle [21]. As can be seen in Table 1, the nanoparticles were smaller than 10 nm; these results agreed with the TEM observations.

**Table 1.** Crystallite size of NPs (calculated from the XRD peak width).

2*θ*	Crystallite size (nm)	Average crystallite size (nm)
44.2	9.3	8.2
51.7	7.1	
76.1	8.1	

To check the presence of NPs in solution (not onto carbon nanoparticles), discharged solution was filtered by syringe filter unit for gas chromatography and mixed with other carbon materials (Kejen black EC600Jd) which do not contain any metal particles. It was then dried and measured by XRD. Figure S1 shows X-ray diffraction patterns of filtered discharged solution, revealing the lack of NP peaks. It is supposed that all sputtered NPs from electrode were loaded on CNBs during discharging.

**Figure 6.**
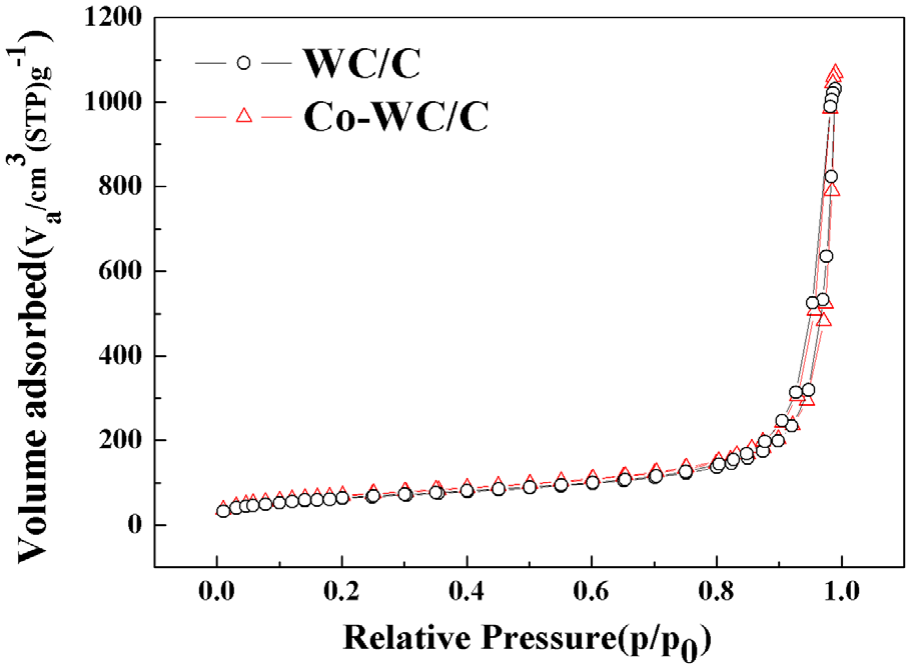
Nitrogen adsorption–desorption isotherms for Co-WC/C.

The pore structure properties of Co-WC/C, including the BET specific surface area, pore volume, and mean pore diameter were obtained by N_2_ adsorption–desorption (Figure 6). The isotherms exhibited type IV and H1 type hysteresis loop characteristics according to the IUPAC classification. [22,23] The total surface area, total pore volume, and mean pore size were 367 m^2^ g^−1^, 1.56 cm^3^ g^−1^, and 8.56 nm, respectively. From these results, it is assumed that the synthesized carbon black presented a highly mesoporous structure; this structure promotes the diffusion of chemical species into the inner region of the carbon, leading to the active use of the catalyst on its surface.

**Figure 7.**
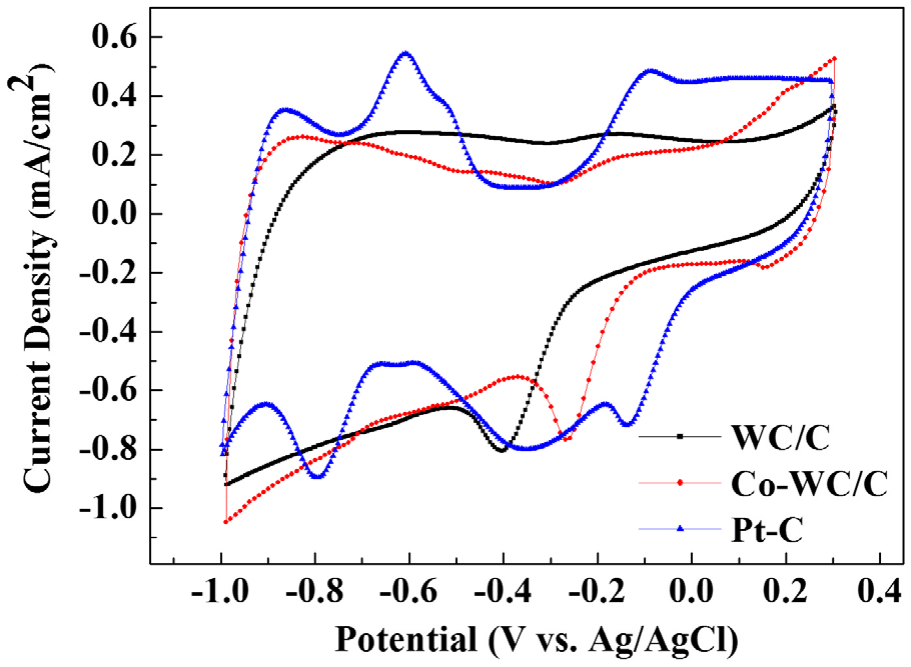
Cyclic voltammetry plots for WC/C, Co-WC/C and Pt/C.

The electrochemical performance of Co-WC/C was evaluated and discussed. Figure 7 shows the cyclic voltammetry (CV) data for the synthesized Co-WC/C, WC/C, and commercial Pt-supported (Pt/C) material under oxygen saturated conditions with 0.1 M KOH electrolyte.

The oxygen reduction potential of Co-WC/C was found to be in the range of –0.09 to –0.35 V. A detailed comparison first focused on WC/C and Co-WC/C. For WC/C, the maximum ORR peak and the on-set potential corresponding to ORR were observed at –0.40 V and –0.23 V, respectively. In the case of Co-WC, the maximum ORR peak and on-set potential were positively shifted to –0.26 V and –0.09 V, respectively. The results of cyclic voltammetry measurements showed that the cobalt nanoparticle promoted WC/C electrocatalyst was very active for ORR, with an onset potential of –0.09 V versus Ag/AgCl, which is over 140 mV better than the value for pure WC/C. However, in comparison with that of the Pt/C catalyst, the ORR activity of Co-WC/C was still incompatible with that of Pt.

**Figure 8.**
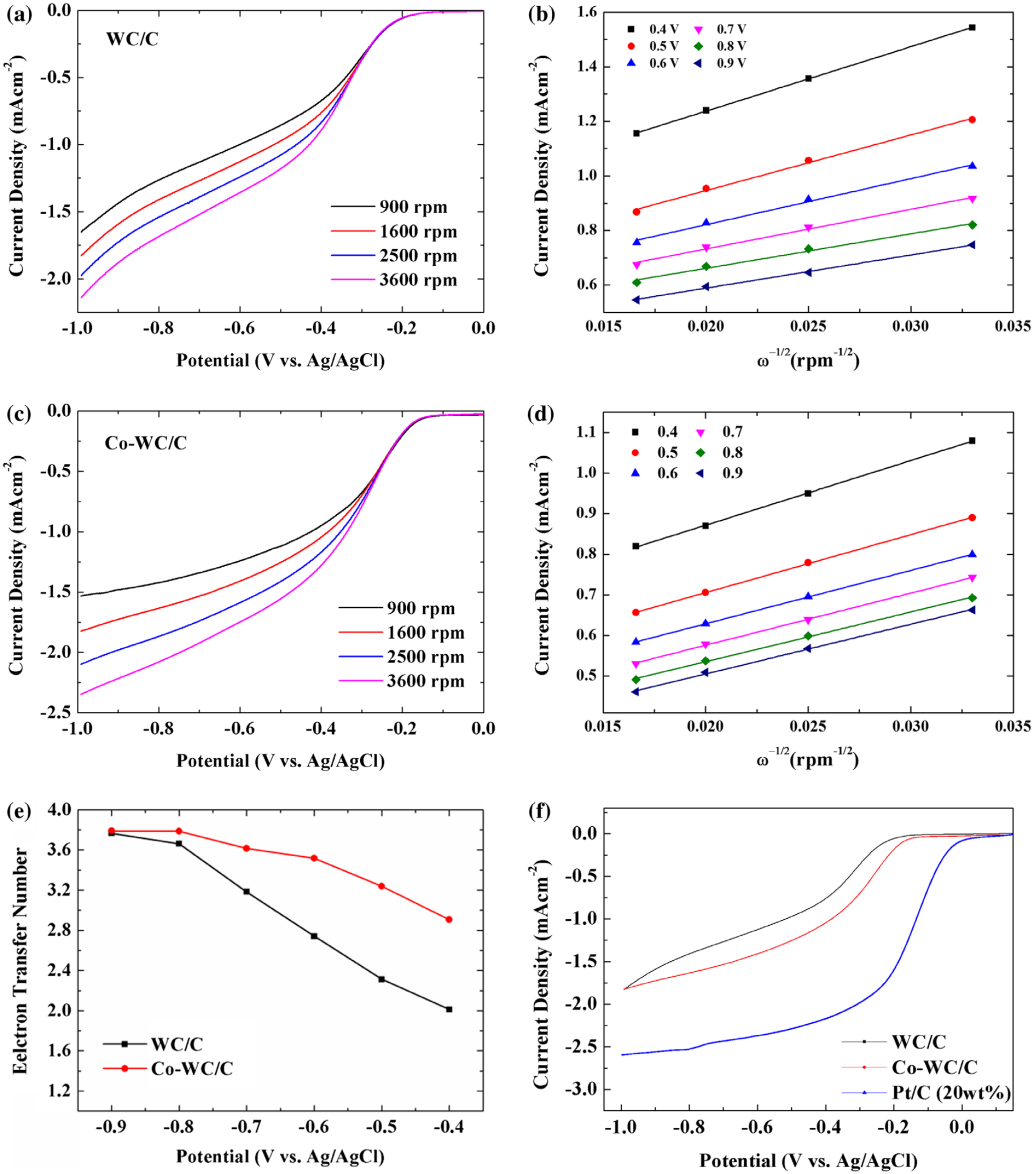
(a, c) Linear sweep voltammetry measurements; (b, d) Koutecky–Levich plot; (e) transferred electron numbers for WC/C and Co-WC/C derived from RDE measurement; (f) linear sweep voltammetry at a rotation rate of 1600 rpm and a scan rate of 10 mV s^−1^.

To gain further insight into the ORR electrochemical process of the Co-WC/C sample, LSV measurements were carried out using a rotating disk electrode (RDE) in O_2_ saturated 0.1 M KOH aqueous solution at a scan rate of 10 mV s^−1^. This was performed at different rotation speeds ranging from 900 to 3600 rpm. Figure 8(a) and (c) display the RDE LSV for WC/C and Co-WC/C. The current density of Co-WC/C was significantly enhanced with increased rotation rate (from 900 to 3600 rpm); this enhancement can be ascribed to the increased diffusion rate at high speeds. The kinetic and diffusion mechanisms of the reaction can be explained by the Koutecky–Levich equations using the obtained parameters [24]:

1/j=1/jL+1/jk

$$1/j=\;1/B\omega^{1/2}+\;1/jk$$

$$B=\;0.62nFCo*Do2/3\nu^{-1/6}$$

where *j* is the measured current density, *j*_*k*_ and *j*_*L*_ are the kinetic and diffusion-limiting current densities, *B* is the Levich current (A), *ω* is the rotation rate of the disk in radians (*ω* = 2π*N*, where *N* is the linear rotation speed), *F* is the Faraday constant (*F* = 96 485 C), *n* is the number of electrons transferred per oxygen molecule, *Co* is the saturated concentration of O_2_ in the electrolyte (1.2 × 10^−6^ mol cm^−3^), *Do* is the diffusion coefficient of O_2_ in the solution (1.9 × 10^−5^ cm^2^ s^−1^), and *ν* is the kinematic viscosity of the electrolyte (1.1 × 10 cm^−2^ s^−1^). The corresponding Koutecky–Levich plots (*j* ^−1^ versus *ω* ^−1/2^) at various electrode potentials (Figure 8(b) and (d)) were obtained from LSV. From the slope of the plot, the number of electrons (*n*) transferred per oxygen molecule of WC/C is calculated to be 2.3 at 0.5 V, suggesting that the ORR process proceeds through a dominant two-electron pathway. The Co-WC/C exhibits the highest n value of 3.23 at 0.5 V. This result indicates that the ORR process on Co-WC/C proceeds preferentially via parallel two-electron and four-electron pathways. The above results reveal that the incorporation of Co nanoparticles into the WC composite improves both the ORR activity and electron transfer in an alkaline medium.

**Figure 9.**
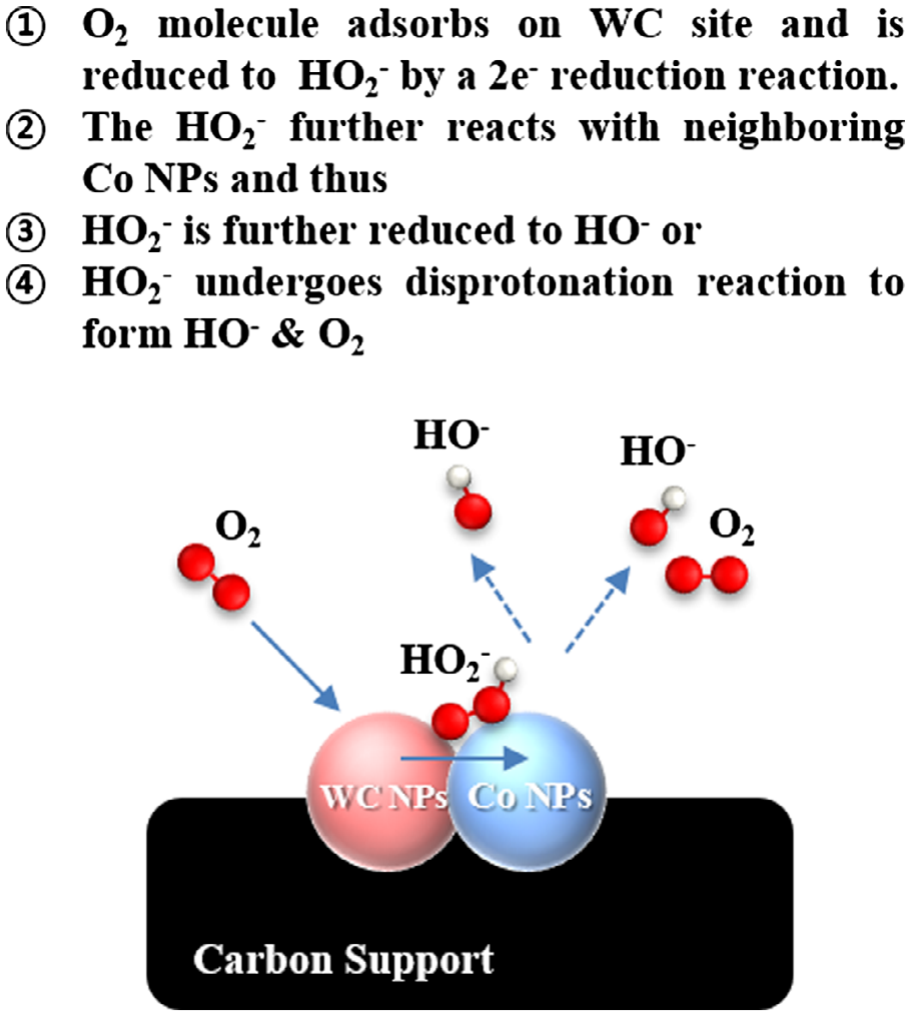
Proposed dual site ORR mechanism on Co-WC/C.

It is predicted that Co nanoparticles neighboring WC/C increase the kinetic property by catalyzing the 2 × 2 serial oxygen reduction reactions in alkaline media (Figure 9). Co addition in WC/C has beneficial effects on ORR activity by formation of active sites such as (1) WC NPs sites, and (2) Co particles, according to dual site mechanism in alkaline media [25].

The comparison of LSV curves at 1600 rpm for WC/C, Co-WC/C, and 20% Pt/C is shown in Figure 8(f). In comparison with WC/C, the half wave potential positively shifted from 0.43 V to 0.32 V in the presence Co NPs, which agreed with the examination from CV. A positive shift in the onset potential points out that the ORR of the Co-WC/C is more favorable than that of WC/C. However, it is still inferior to the state-of-art commercial 20% Pt/C in terms of both the onset potential and current density.

**Figure 10.**
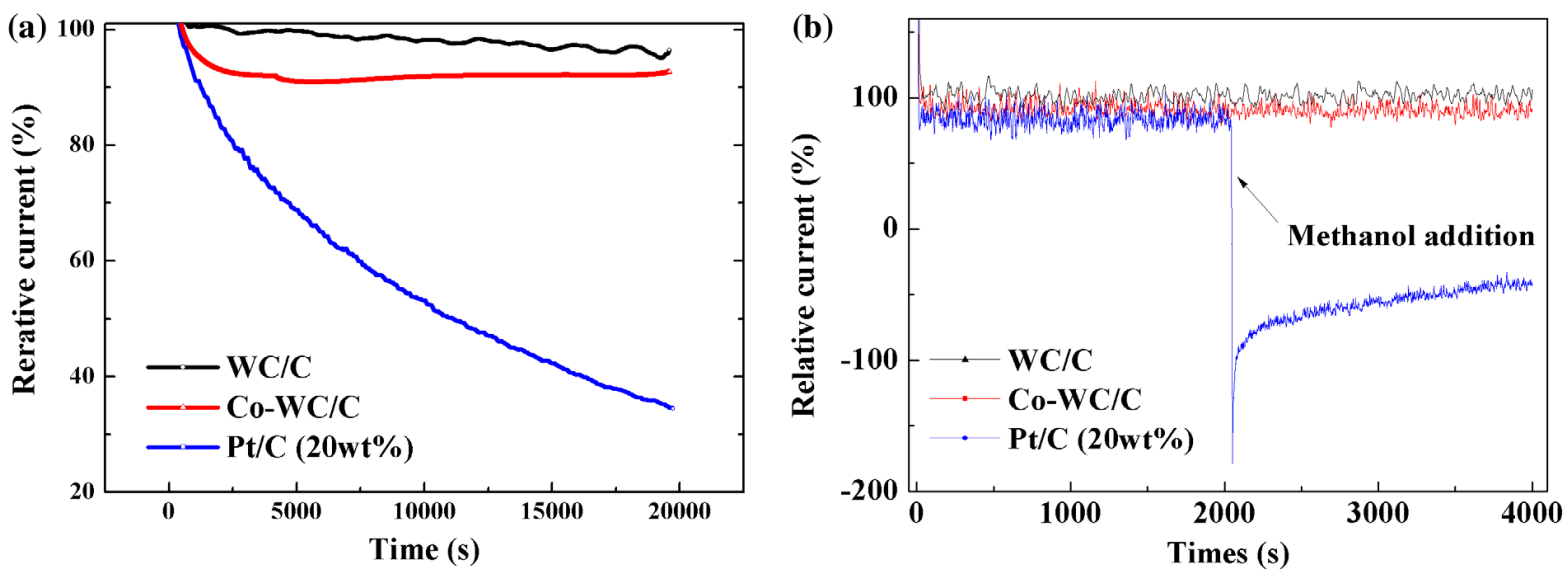
Chronoamperometric responses of Co-WC/C compared with that of Pt/C catalyst in O_2_-saturated 0.1 M KOH at –0.4 V (a) without and (b) with methanol.

Another important property of catalysts for current fuel cell applications is their durability in long-term operation. Figure 10(a) shows the stability of WC/C, Co-WC/C, and commercial Pt/C catalysts, which were tested at –0.4 V (mass transport region) for 20,000 s in O_2_ saturated 0.1 M KOH solution at a rotation speed of 1600 rpm. Evidently, WC/C and Co-WC/C showed a high durability for long-term operation after continuous reaction for 20,000 s, with relative current remaining at over 90% of the initial current. On the other hand, an obvious decrease in current was observed for the commercial Pt/C catalyst. To further assess the tolerance to the methanol crossover effect, 3 M methanol (CH_3_OH, purity 99.8%, Kanto Chemical) was introduced at 2000 s into an O_2_-saturated 0.1 M KOH solution during the chronoamperometric measurement at –0.4 V (Figure 10(b)). After the introduction of 3 M methanol, commercial Pt/C showed an obvious current drop corresponding to the methanol oxidation reaction, whereas that of WC/C and Co-WC/C remains unchanged and stable without degradation. From these results, we can conclude that Co-WC/C nanocomposites presented a higher durability than that of commercial Pt/C catalysts in an alkaline medium.

## 4. Conclusions

Co-WC/C nanocomposites were first synthesized by SPP as non-precious metal electrocatalysts for ORR. Electrochemical measurements in an alkaline medium revealed that the Co NPs affected the ORR activity. The introduction of Co NPs led to not only superior ORR activity in terms of onset potential and peak potential, but also to a more efficient electron transfer process compared to that of pure WC/C. Compared to commercial 20% Pt/C, Co-WC/C also exhibits long-term durability for ORR. Therefore, we believe that the introduction of Co NPs is an effective method for enhancing the ORR activity of pure WC; also, Co-WC/C could be a promising alternative ORR catalyst for application to alkaline fuel cell devices. In addition, SPP shows a potential that can be extended to the synthesis of other non-noble metal nanoparticles simply by changing the electrodes. We believe that further development using SPP in this field will facilitate and accelerate the application of non-noble metal ORR electrocatalysts for advanced energy conversion devices.

## Notes on Contributors

***Jun Kang*** is an assistant professor and received his doctor of Engineering. The author's research interests include Nano Materials, Carbon Materials, and Plasma in Liquid. His recent publications include Synthesis of structure-controlled carbon nano spheres by solution plasma process. Carbon. 2013;60:292–298; Hierarchical meso–macro structure porous carbon black as electrode materials in Li–air battery. J Power Sources. 2014;261:156–161; A simple synthesis method for nano-metal catalyst supported on mesoporous carbon: the solution plasma process. Nanoscale. 2013;5:6874– 6882; In situ solution plasma synthesis of mesoporous nanocarbon-supported bimetallic nanoparticles RSC Advances 2015 ; 5(37): 29131-29134.

***Hye-Min Kim*** is a doctoral student. The author's research interests include Carbon Material and Metal Oxide The author's recent publication includes Synthesis of colloidal MnO 2 with a sheet-like structure by one-pot plasma discharge in permanganate aqueous solution. RSC Advances. 2016; 6: 2826-2834.

***Nagahiro Saito*** is a full professor and received his doctor of Engineering. The author's research interests include Plasma in Liquid and Nano materals. His recent publications are Synthesis process of gold nanoparticles in solution plasma. Thin Solid Films. 2009; 518 (3), 912-917; Size-controlled gold nanoparticles synthesized in solution plasma. The Journal of Physical Chemistry C. 2011; 115 (50), 24569-24576.

***Myeong-Hoon Leeis*** a full professor and received his doctor of Engineering. The author's research interests are Electrochemistry, Corrosion control and Surface coating. His recent publications are Influence of annealing temperatures on corrosion resistance of magnesium thin film-coated electro-galvanized steel Modern Physics Letters B. 2015 ; 29,1540015-1~1540015-5; Polarization characteristics of four types of coating films by thermal spray in seawater solution. Modern Physics Letters B. 2015; 29, 1540019-1~1540019-6.

## Disclosure statement

No potential conflict of interest was reported by the authors.
